# Factors promoting and limiting antimicrobial resistance in the environment – Existing knowledge gaps

**DOI:** 10.3389/fmicb.2022.992268

**Published:** 2022-09-20

**Authors:** Agata Goryluk-Salmonowicz, Magdalena Popowska

**Affiliations:** Department of Bacterial Physiology, Institute of Microbiology, Faculty of Biology, University of Warsaw, Warsaw, Poland

**Keywords:** antibiotics, antibiotic resistance dissemination, bacteriophages, non-clinical resistance reservoirs, strategies to combat drug resistance

## Abstract

The dissemination of multidrug-resistant bacteria strains and genes carrying antibiotic resistance is currently considered to be one of the most important global problem. The WHO calls for the need to contain the spread of Antimicrobial Resistance (AMR) from all possible sources. There have been many international actions grouping scientists studying this phenomenon, and quite a lot of scientific projects devoted to this problem have already been carried out. As well, so far several strategies have been developed that can inhibit the AMR spread. In this mini-review, we highlight overlooked aspects that seem to be crucial for creating a comprehensive picture of AMR, especially in the context of One Health approach.

## Introduction

Spread of Antibiotic Resistant Bacteria (ARB) and Antibiotic Resistant Genes (ARGs) in the environment is now recognized as one of the top 10 global public health threats for humanity. In 2019 alone, about 5 million people worldwide died due to infections with multidrug resistant bacteria while undergoing treatment for various other diseases, and over a million people died after being infected with multidrug resistant bacteria. WHO calls for global action to counter dissemination of antibiotic resistance. Antimicrobial resistance (AMR) has become one of the main issues in the One Health strategy announced by the WHO. The One Health strategy recommends environmental monitoring and joint research carried out by specialists in public health, veterinary and environmental protection to stop the spread of AMR. So far, several strategies have been developed to inhibit the AMR spread. One of them is the Joint Programming Initiative on Antimicrobial Resistance, JPIAMR, launched in 2014. To date, JPIAMR has supported 99 projects and 38 networks gathering over 1,400 researchers by investing 125 million euro^1^. JPIAMR is a global collaborative organization of 29 nations acting in accordance with One Health approach. The JPIAMR priority topics are diagnostics, environment, interventions, surveillance, therapeutics and transmission. Two of them are closely related to fate of AMR in the environment, namely “Transmission” and “Environment.” The “Transmission” aspect covers understanding and preventing AMR transmission, while the “Environment” topic deals with explaining the role of environment in persistence, selection and spread of AMR. By the end of 2021 JPIAMR supported 79 projects related to these two topics (see text footnote 1). Most of those projects were aimed at monitoring emerging pathogens in hospitals and their transmission into the environment (22 projects). Some other projects were focused on detecting ARB and ARGs among WWTP, livestock or aquaculture (20 projects), while 14 projects were aimed at developing new technologies to eliminate ARB or ARGs from manure, agricultural animals, aquaculture or water environments. Among all these projects, none aimed at explaining the role of the environment in the spread of AMR. Some factors promoting AMR spread are known, but plenty of determinants are still not well-understood. One of them is the impact of biodiversity on the antibiotic resistance spread. To explain the role of environment in AMR spread, an international project entitled ‘ANTIVERSA – Biodiversity as an ecological barrier for the spread of clinically relevant antibiotic resistance in the environment’ was launched. The project is supported by the Biodiversa European Partnership. In this mini-review, we are focusing on factors that influence AMR spread but are not commonly researched. To explain the role of the environmental aspects in the persistence, selection and spread of AMR a holistic approach should be taken.

## Factors promoting antimicrobial resistance dissemination

The most obvious factor that promotes AMR spread in the environment are antibiotics responsible for the selection pressure (Table 1). Enormous use of antibiotics to treat human and animal diseases has been reported worldwide. Until recently, antibiotics were also commonly used for animal growth promotion (Dibner and Richards, 2005; Castanon, 2007; Bengtsson-Palme et al., 2018; Muurinen et al., 2021). Government regulations and prohibitions of antibiotic use as growth promoters appeared scarcely in 2006 in the European Union, in 2017 in the United States and in 2020 in China (Dibner and Richards, 2005; Centner, 2016; Hu and Cowling, 2020). Over 80% of antibiotics consumed by humans or animals end up in feces in sewage treatment plants. Widespread presence of antibiotics leads to the acquisition of resistance determinants by bacteria inhabiting different environments and promotes incidence of ARB and ARGs (Kobashi et al., 2007; Santamaría et al., 2011; von Wintersdorff et al., 2016; Pérez-Valera et al., 2019). Despite modern technologies and substantial removal of resistant bacteria from the water fraction, digested sludge or treated wastewater still contain heightened levels of ARB and ARGs (Parsley et al., 2010; Gatica and Cytryn, 2013; Rizzo et al., 2013; Balcazar, 2014; Calero-Cáceres et al., 2014). Using this material as biofertilizer on agricultural fields, causes AMR spread in the soil and aqueous systems (Finley et al., 2013; Yang et al., 2014; Ross and Topp, 2015; Qiao et al., 2018; Pérez-Valera et al., 2019; Krzemiński and Popowska, 2020; Do et al., 2022). Many researchers showed also that direct application of animal manure to agricultural land, introduces ARB and ARGs to the environment (Kyselková et al., 2015; Wei et al., 2016; Muziasari et al., 2017; Berendsen et al., 2018; Guo et al., 2018; Pérez-Valera et al., 2019; Do et al., 2022). Kyselková et al. (2015) confirmed that ARG such as *tet*O, *tet*W, *tet*Q and *intl*1 persisted in soil manured with cattle feces, Pérez-Valera et al. (2019) showed abundance of *tet*Y genes in the same type of soil while Guo et al. (2018) and Do et al. (2022) isolated different ARGs genes from pig manure amended soil. Moreover, aquaculture was confirmed as a source of ARG. Muziasari et al. (2017) detected 13 different ARG from farmed fish feces determining resistant to aminoglycosides, chloramphenicol, tetracyclines, trimethoprim and sulfonamide.

**TABLE 1:** Factors that promote AMR dissemination.

	Factor	Mechanism responsible for AMR spread	Action promoting AMR spread by the factor	Reason of occurrence of the factor in the environment
1	Antibiotics	Mutations of DNA caused by selection pressure	Contamination with antibiotics	Usage in agriculture, aquaculture, medicine, veterinary and industry
2	ARB	HGT	Contamination with ARB/ARGs	Soil fertilization with manure, biosolids or treated wastewater
3	ARGs	HGT	Contamination with ARB/ARGs	Soil fertilization with manure, biosolids or treated wastewater
4	Bacteriophages	HGT	Contamination with ARB/ARGs	Soil fertilization with manure, biosolids or treated wastewater
5	Heavy metals	Co-selection	Contamination with heavy metals	Usage in agriculture, medicine, veterinary and industry
6	Microplastic	Sorption process of antibiotics, heavy metals and ARB; Co-selection; HGT	Contamination with ARB/ARGs, heavy metals, proliferation of ARB	Usage in agriculture, medicine, veterinary and industry
7	Organic carbon content above 5%	HGT	Proliferation of ARB	Soil fertilization with manure, biosolids or treated wastewater
8	Temperature above 30°C	HGT	Proliferation of ARB	Global warming

ARB, Antibiotic resistant bacteria; ARGs, Antibiotic resistance genes; ARB/ARGs, Antibiotic resistant bacteria or Antibiotic resistance genes; HGT, Horizontal gene transfer.

Nowadays, ARB and ARGs are considered as very important factors promoting AMR (Table 1). Many research projects showed that mobile genetic elements, like plasmids, transposons or integrons, act as genetic vehicles to spread ARGs between cells by horizontal gene transfer (HGT) (Xian-Gang et al., 2008; Finley et al., 2013; Yang et al., 2014; Ross and Topp, 2015; Qiao et al., 2018; Pérez-Valera et al., 2019; Piotrowska et al., 2020; Zalewska and Popowska, 2020). Interestingly, it has been confirmed that bacteriophages contain ARGs and can also spread them (Witte, 2004; Muniesa et al., 2004; Brabban et al., 2005). First laboratory experiments on bacteriophages containing ARGs showed that they can transduce genes conferring resistance to aztreonam, ceftazidime, and imipenem in *Pseudomonas aeruginosa*, methicillin in *Staphylococcus epidermidis* and tetracycline in *Staphylococcus aureus* and *Actinobacillus actinomycetemcomitans* (Blanchard et al., 1986; Blahova et al., 1993; Pereira et al., 1997; Willi et al., 1997). Further research confirmed that ARGs were detected in bacteriophages isolated from human feces and fecally polluted environments. The most abundant ARGs detected in phage fraction from human stool samples were *bla*_TEM_, *bla*_CTX–M_, *arm*A and *qnr*A, while in wastewater *bla*_TEM_ and *mec*A were commonly detected (Colomer-Lluch et al., 2011a,b; Quirós et al., 2014). Studies have demonstrated that the phageome of other environments also harbor ARGs. Ross and Topp (2015) confirmed that soilborne bacteriophages serve as a reservoir of ARGs in soils (*aad*A, *str*A, *str*B, and *sul*1 genes), while Colomer-Lluch et al. (2011b) identified ARGs in phage DNA isolated from river water (*bla*_TEM_, *bla*_*CTX–M*_, *mec*A). Moreover, research showed that municipal biosolid-derived bacteriophages play a significant role in the transduction of resistance to cefoxitin and sulfamethazine. Transduction process was observed in an experiment performed with coliform bacteria isolated from agricultural soil incubated in the presence of subclinical concentrations of antibiotics and biosolid-derived bacteriophages (Ross and Topp, 2015). Other studies confirmed that ARGs from phage DNA were transferred to *E. coli* recipient hosts in laboratory conditions (Colomer-Lluch et al., 2011b). Despite reports on the ability of phage-encoded ARGs to confer antibiotics resistance to bacteria, the contribution of bacteriophages to AMR spread in the environment is little discussed. Bacteriophages should be considered as an important factor promoting the spread of AMR (Table 1).

Another serious factor that should be considered as resistance promoter are heavy metals (Table 1). Heavy metals, especially cadmium (Cd), copper (Cu), lead (Pb), mercury (Hg) and zinc (Zn), are commonly used in agricultural and aquacultural practices, resulting in heavy metals being transferred to the environment and causing AMR dissemination through the co-selection phenomenon. The co-selection occurs when different resistance genes are located on the same mobile genetic element (co-resistance), or when one genetic determinant generate resistance to more than one stressor (cross resistance) (Chapman, 2003; Seiler et al., 2012; Goryluk-Salmonowicz and Popowska, 2019; Imran et al., 2019). Numerous studies confirmed the correlation between elevated heavy metal concentrations and increased phenotypic or genotypic antibiotic resistance. The co-selection of metal driven antibiotic resistance in bacteria has been observed in many environments, such as marine environment, soil environment, manure, sediments or drinking water (McArthur and Tuckfield, 2000; Berg et al., 2005, 2010; Stepanauskas et al., 2006; Wright et al., 2006; Graham et al., 2011; Davin-Regli, 2012; Hölzel et al., 2012; Seiler et al., 2012; Yazdankhah et al., 2018; Zhang et al., 2018; Imran et al., 2019). For example, Stepanauskas et al. (2006) performed an experiment in freshwater microcosms amended with Cd, Ni, ampicilin or tetracycline, and his results showed that frequency of antibiotic resistance were elevated in metal amended microcosms, while Zhang et al. (2018) confirmed that copper at the levels of 10 and 100 mg/L significantly increased water bacteria resistance to erythromycin, kanamycin and rifampin.

Interestingly, recently published research indicated microplastic particles (MPs) as a new factor promoting antibiotic resistance spread (Table 1; Bowley et al., 2021; Guo et al., 2020; Radisic et al., 2020). The presence of microbial communities on the plastic surfaces were detected for the first time by Zettler et al. (2013). MPs have hydrophobic surfaces and are easily colonized by microbial biofilms. Moreover the sorption process of antibiotics and heavy metals occurs on MPs (Godoy et al., 2019; Mammo et al., 2020; Wang et al., 2020). MPs were detected as a vector for the proliferation of heavy metal and antibiotic resistant bacteria, and HGT between microorganisms presented on microplastics was confirmed (Imran et al., 2019; Oberbeckmann et al., 2018; Rummel et al., 2017). Zhang et al. (2020) showed the presence of multidrug-resistant *Vibrio* species on marine MPs, Song et al. (2020) isolated *Escherichia coli* from marine MPs while Yang et al. (2020) confirmed that MPs isolated from soil contained higher relative abundance of the pathogenic bacteria *Acinetobacter johnsonii* and *E. coli*. Many authors have isolated ARGs from MPs sampled from wastewater treatment plants, aquatic, terrestrial, and air environments (Su et al., 2021; Sun et al., 2021; Wang et al., 2020; Wu et al., 2019; Yang et al., 2020). Clinically important ARGs, like *sul*1, *str*B, *tet*A, *tet*C, *tet*X, *erm*B, *erm*E, *aac*(3), *mac*B, and *bla*_TEM_ were detected on MPs in freshwaters and sea waters (Alcock et al., 2020; Shi et al., 2020; Wang et al., 2020). Lu X. M. et al. (2020) have detected more than 30 different ARGs on the surface of MPs collected from vegetable soil (Lu X. M. et al., 2020). Although MPs have been shown to contain ARB and ARGs, better understanding the role of this factor in the spread of AMR is needed.

One more group of factors responsible for AMR dissemination should be considered, namely soil properties and cultivation conditions. Lu W. et al. (2020) examined ARGs spread by plasmid mobilization in different agricultural soil types and under different conditions. Experiments were performed in soil microcosms spiked with ARB where ARGs were detected by Real-Time PCR. Five soil types were used: loam soil (35% of silt), loamy sand soil (9% of silt), sandy loam soil (15% of silt) and sandy clay loam soil (21% of silt). Research revealed that the frequency of plasmid mobilization and expression of ARGs was highest in the loam soil. Loam soil contains rich nutrients, more habitable pore-space, and retains moisture, so it is considered as creating beneficial conditions for microorganism activity (Scherr et al., 2007; Djokic et al., 2013). Moreover, the impact of soil depth on ARGs spread was detected. Topsoil and soil from deeper layers didn’t promote ARGs spread, while the highest ARGs level was observed at the depth of 10 – 15 cm. Obtained results are consistent with other studies and can be explained by natural microbe activity in the soil (Debasmita et al., 2011; Wang et al., 2014). Two more factors impacting ARGs spread were detected by Lu W. et al. (2020), namely temperature and nutrient content. High temperature (above 30°C) and high level of nutrients promoted ARGs spread (Table 1). To sum up, soil properties, soil depth and temperature are important factors for AMR spread in the environment. However, little attention is paid to soil properties as a factor promoting AMR dissemination. Interestingly, rhizosphere and some of its types were also shown to promote ARGs spread (Lu W. et al., 2020). Results showed that ARGs transfer and ARGs abundance was higher in maize rhizosphere than tomato or wheat rhizosphere soils.

## Factors inhibiting antimicrobial resistance dissemination

One of the widely researched factors known to limit AMR spread is microbial community inhabiting soil. It has been demonstrated that soil microbial diversity is a key factor in controlling the success of invasion by ARB (van Elsas et al., 2007, 2012). Van Elsas showed that the survival of *E. coli* O157:H7 derivate strain added as invader to soil microcosms in laboratory conditions depended on the amount of soil bacteria. That strain was resistant to rifampicin (chromosomal mutation) and kanamycin (ARG on MGE). In his study published in 2007, he used soil fumigation technique to obtain microcosms with modified microbial communities. Different depths of fumigation were applied, from 0h to 24 h. A progressive decline in numbers of invader was observed in unfumigated soil (from the initial log 7.2 down to the detection threshold), while the survival of ARB in all fumigated soils was significantly enhanced. Similar results were obtained in research published in 2012 by van Elsas et al. where microcosms of sterile soil inoculated with a gradient of diversity present in serially diluted natural soil was used. The author noticed that among soil native microorganisms, bacteria with typical actinobacterial morphology were dominant. It is worth noting that other studies clearly showed that soil was markedly enriched in *Actinobacteria* after treatment with manure (Chen et al., 2017). Chen et al. (2017) conducted microcosm experiments where soils were amended with pig manure (irradiated soil or fresh soil), and then diversity of bacterial communities was examined. Bacterial communities from all the samples were classified into different clusters based on PCoA analysis. The results indicated that manure application shifted the bacterial composition in tested soils and that *Actinobacteria* could limit ARGs spread, as the abundance of those genes in soil amended with manure was significantly lower than in sterile soil with manure. The dilution-to-extinction method was also used by other authors (Matos et al., 2005; Wertz et al., 2006, 2007). Results confirmed previous findings, clearly indicating a negative correlation between the diversity of soil microbiota and survival of the invader. Nevertheless, it was not clear whether ARB decline was due to antagonism interactions or competition for nutrients. It was hypothesized that microbial communities with lower metabolic diversity might be more prone to invasion by alien species than those using wide range of substrates. Interestingly, further research demonstrated that some bacteria taxa of indigenous soil microorganisms can promote AMR (Chee-Sanford et al., 2009; Pérez-Valera et al., 2019; Lu X. M. et al., 2020). Two soil bacteria genera, namely *Dyella* sp. and *Variovorax* sp., were confirmed to capture and keep TET-resistance genes acting as reservoirs of ARG (Kobashi et al., 2007; Santamaría et al., 2011; Pérez-Valera et al., 2019). To sum up all the results, it was confirmed that some native soil microorganisms hinder soil invasion with ARB and ARGs, but there are also a few genera that can promote dissemination of AMR. If those bacteria start to dominate in the environment they can be considered as factors promoting AMR.

As for now, since environmental factors limiting AMR spread are not clear, research work is focused on developing new technologies to eliminate ARB and ARGs. Regarding soil environments, many studies showed that manure composting is an effective method to reduce ARB and should be performed as preapplication treatment (Ross and Topp, 2015; Do et al., 2022). Moreover, recent studies examined the dynamics of microbial communities when pig slurry was subjected to different treatments, such as storage for 4 months, composting for 2 months and anaerobic digestion for 3 months (Do et al., 2022). The results showed that all treatment strategies were effective in reduction of bacterial pathogens. Anaerobic digestion is an optimal pig slurry treatment to reduce ARGs and MGEs. Other studies compared pig manure storage and composting treatments and concluded that composting process is more effective than storage in reducing microbial loads, ARGs and MGE (Zalewska et al., 2022). Both works emphasized that both slurry and pig manure, after appropriate treatment, can be part of a natural, safe soil fertilization strategy. Regarding water environments, different filtration systems are under investigations, for example, membranes coated with antibacterial titania-silica-core nanoparticles for inactivating ARB, or phage-wetland combined technologies to reduce ARB and ARGs. The aim of these new technologies is to eliminate contaminants of emerging concern (CECs).

## Discussion

Studies suggest that indigenous environmental microorganisms combat invader ARB that are not well adapted to the natural environment. The question is what bacterial taxa are best suited for limiting ARGs? Can we determine native microbial communities that are least susceptible to HGT? It has been reported that some manure-borne bacteria have better ability to adapt to the soil environment and disseminate ARGs. Identification of bacteria taxa that pose the biggest risk for ARGs dissemination is a priority now. It is suggested that those taxa of high risk could even replace native soil microorganisms. Little is known about the contribution of bacteriophages to the spread of ARGs in the environment. Recent studies confirmed a significant role of phages in the transduction of ARGs. Interestingly, manure processing (digestion, dewatering or composting) or incubation time had no effect on ARG abundance in bacteriophages fraction. It is unclear why ARGs abundance in phageome neither increase after fertilization with manure nor decrease after manure treatment. Nevertheless, many on-going JPIAMR projects are focused on the use of bacteriophages to combat AMR, but it can be a big challenge to develop such environmentally safe technology. The existing gaps in knowledge about the impact of soil or water physico-chemical properties on AMR spread are also noteworthy. Recent research studies confirmed the correlation between soil type and soil properties, and ARGs abundance. Further studies with more comprehensive approaches will provide more detailed information about the factors promoting and limiting AMR spread.

## Conclusion

Natural ecosystems show variable resistance to invasion by ARB. It depends on many factors that should be taken into account in their entirety. Without proper understanding of the role of environmental factors that promote AMR spread our efforts will be useless. We have to take into account all the factors connected with AMR spread rather than focus only on any one of them. Regarding soil environment for example, not only biodiversity is important but also soil type, soil properties and cultivated crops. It seems that fertilizers (mineral or natural) as well as used chemical plant protection agents are also important.

Thus, comprehensive, interdisciplinary research studies are lacking that could provide broad results on dissemination of AMR in the environment, conducive to introducing new standards by the European Commission or governments of individual countries, concerning, e.g., the use of antibiotics in veterinary medicine, agriculture or animal farming and methods of wastewater treatment.

## Author contributions

AG-S: investigation, formal analysis, writing – original draft, and visualization. MP: conceptualization, writing – review and editing, supervision, funding acquisition, and project administration. Both authors contributed to the article and approved the submitted version.

## Footnotes

1
https://www.jpiamr.eu/
